# Regression Discontinuity for Causal Effect Estimation in Epidemiology

**DOI:** 10.1007/s40471-016-0080-x

**Published:** 2016-08-05

**Authors:** Catherine E. Oldenburg, Ellen Moscoe, Till Bärnighausen

**Affiliations:** 1 Department of Epidemiology, Harvard T.H. Chan School of Public Health, 665 Huntington Avenue, Boston, MA USA; 2 Department of Global Health and Population, Harvard T.H. Chan School of Public Health, 665 Huntington Avenue, Boston, MA USA; 3 Africa Centre for Population Health, PO Box 198, 3935 Mtubatuba, South Africa; 4 Institute of Public Health, University of Heidelberg, Heidelberg, Germany

**Keywords:** Regression discontinuity, Causal inference, Quasi-experimental, Epidemiologic methods, Econometrics

## Abstract

Regression discontinuity analyses can generate estimates of the causal effects of an exposure when a continuously measured variable is used to assign the exposure to individuals based on a threshold rule. Individuals just above the threshold are expected to be similar in their distribution of measured and unmeasured baseline covariates to individuals just below the threshold, resulting in exchangeability. At the threshold exchangeability is guaranteed if there is random variation in the continuous assignment variable, e.g., due to random measurement error. Under exchangeability, causal effects can be identified at the threshold. The regression discontinuity intention-to-treat (RD-ITT) effect on an outcome can be estimated as the difference in the outcome between individuals just above (or below) versus just below (or above) the threshold. This effect is analogous to the ITT effect in a randomized controlled trial. Instrumental variable methods can be used to estimate the effect of exposure itself utilizing the threshold as the instrument. We review the recent epidemiologic literature reporting regression discontinuity studies and find that while regression discontinuity designs are beginning to be utilized in a variety of applications in epidemiology, they are still relatively rare, and analytic and reporting practices vary. Regression discontinuity has the potential to greatly contribute to the evidence base in epidemiology, in particular on the real-life and long-term effects and side-effects of medical treatments that are provided based on threshold rules – such as treatments for low birth weight, hypertension or diabetes.

## Introduction

Randomized controlled trials are typically considered the gold standard for causal inference. Although most randomized controlled trials are designed to elicit unbiased estimates of efficacy under ideal trial conditions, this may be at the expense of external validity. For example, eligibility criteria in trials may be designed to minimize loss to follow-up but may lead to samples of patients that are not representative of the broader population of patients who may benefit from treatment. Other design features of trials may limit generalizability of results. For example, a landmark trial of HIV transmission following antiretroviral therapy (ART) uptake required enrolled couples to be mutually disclosed, monogamous, and stable, limiting the generalizability of the trial results to other relationship structures [1]. Furthermore, randomized controlled trials are frequently infeasible or unethical, for example, in situations where the exposure cannot be randomized or in the absence of equipoise. Epidemiologists are therefore frequently tasked with the identification of causal effects from observational data. The most commonly used methods, such as multiple regression, matching, or probability weighting, rely on the untestable assumption of no unmeasured confounding. Although this assumption may be reasonable if detailed data on numerous covariates can be collected, this is not possible in many situations, and these analyses may still be biased by unmeasured and uncontrolled confounding of the exposure-outcome relationship.

Quasi-experimental methods are a class of methods that take advantage of exogenous sources of variation in exposure assignment to emulate random treatment assignment, as in a randomized controlled trial [2, 3]. An exogenous source of variation, called the instrument in instrumental variable analyses, is a variable that is a cause of the exposure but is otherwise unrelated to the outcome and, as such, is outside or ‘exogenous’ to the causal structure under study. Examples include implementation of policies (e.g., China’s one-child policy [4]), calendar time (e.g., time trends in street lighting coverage [5]), or genotypes determining phenotypes (i.e., Mendelian randomization) [6, 7]. By virtue of their use of an exogenous source of variation, quasi-experiments do not require the assumption of no unmeasured confounding and, thus, may be advantageous in scenarios where this assumption is unlikely to hold.

The regression discontinuity design is a quasi-experimental approach that was first described in 1960 in the educational psychology literature [8]. The regression discontinuity design can be thought of as an extension of instrumental variable analysis, in circumstances where an exogenous source of variation arises from a continuously measured random variable that at least partially assigns treatment or other exposure based on a threshold rule [9]. For example, prostate-specific antigen (PSA) level has recently been utilized as an assignment variable for determining eligibility for further prostate cancer workup, as men with a PSA of over 4.0 ng/mL are eligible for further screening and workup [10••]. If the assignment variable determining treatment is a cause of treatment and is not independently correlated with the outcome, then – in a small range of values around the threshold that determines treatment – patients on each side of the threshold are expected to be similar with respect to all prognostic characteristics for the outcome. Thus, all baseline covariates are expected to be balanced between the two groups, as in a randomized controlled trial where randomization guarantees that the exposure is assigned exogenously.

Despite the considerable potential advantages that the regression discontinuity design has for the identification of causal effects in the absence of randomized controlled trials, their application remains relatively uncommon in the epidemiologic literature [11•, 12•]. Here, we review the regression discontinuity design, the assumptions for estimation of causal effects, and the estimation of complier average causal effects using instrumental variable methods. We conclude by reviewing recent applications of regression discontinuity designs in epidemiology.

## The Regression Discontinuity Design

**Fig. 1.**
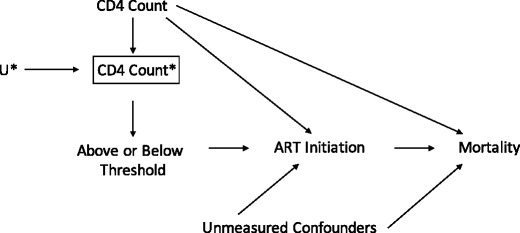
An illustration of the regression discontinuity design using a directed acyclic graph. Directed acyclic graph (DAG) illustrating the regression discontinuity for the example of ART initiation and mortality. CD4 count is the assignment variable, which is measured with error (depicted by the *asterisk*), which is used to determine whether a patient is above or below the threshold, and thus eligible for treatment. CD4 Count* (CD4 count measured with error) is in a box to depict that the analysis is restricted to only patients who are immediately above and below the threshold. The DAG depicts that there are no open backdoor paths between mortality and eligibility for treatment based on the threshold, even in the presence of unmeasured confounding of ART status and mortality

Regression discontinuity designs can be applied in situations where a threshold rule is used to determine treatment assignment. In this situation, a continuous variable (the assignment variable) is used, at least in part, to determine whether or not an individual is assigned to a treatment. In the presence of random variability in the assignment variable, patients immediately above and below the threshold are expected to be similar with respect to the distribution of all baseline covariates and, thus, are expected to be exchangeable. Such random variation can arise due to measurement error of biomarkers, sampling variability, or other sources of variation that are unrelated to the exposure and the outcome. In the regression discontinuity design, the threshold rule represents an exogenous source of variation in (or the instrument for) treatment assignment. The exchangeability assumption in regression discontinuity is also often called the *continuity assumption*. Figure 1 illustrates the causal structure in the regression discontinuity case in a directed acyclic graph.

In the potential outcomes framework of causal inference, the primary challenge for identification of effects is identification of the counterfactual [13]. In the binary treatment case, identification of the individual counterfactual outcomes would require observing the same individual’s outcome under two different treatment scenarios, which is impossible in most cases. Average causal effects can be identified, however, under certain conditions when the population is on average exchangeable with a population under a different treatment status. If the continuity, or exchangeability, assumption holds in a regression discontinuity study, then individuals whose measured values are immediately below the threshold can serve as a valid counterfactual for those immediately above the threshold, as the distribution of baseline covariates is expected to be the same in these two groups.

### Randomized Controlled Trials in a Regression Discontinuity Framework

When the assignment variable is a random number that is generated by the researcher, the regression discontinuity design is equivalent to a randomized controlled trial [14]. For example, if the assignment variable *Z* follows a uniform distribution over the range [0, 10], patients who are randomly assigned a value of *Z* of ≥5 receive treatment, whereas those who assigned a value of <5 do not. In this case, the assignment variable is the random number *Z* and the threshold is 5. When *Z* is randomly assigned, it is independent of potential outcomes, and thus, the continuity assumption will hold. When treatment is randomly assigned, and randomization is successful, all patients on each side of the threshold will be exchangeable. Of note, in randomized controlled trials, unlike in most regression discontinuity designs, the effect estimated is an average effect in all patients in the analytic sample. In this example, the causal effect of treatment can therefore be estimated as the difference or ratio between *E*[*Y*|*Z*≥5] and *E*[*Y*|*Z*<5], which is the ITT effect commonly estimated in randomized controlled trials.

### Sharp and Fuzzy Regression Discontinuity Designs

**Fig. 2:**
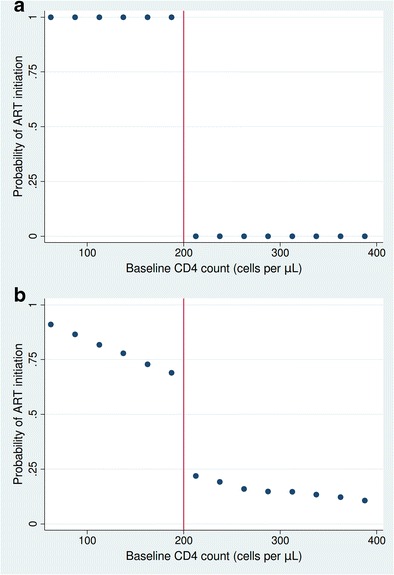
. Illustration of the 'sharp' and the 'fuzzy' regression discontinuity design. Hypothetical scenario depicting treatment assignment in the sharp or deterministic (**a**) and fuzzy or probabilistic (**b**) regression discontinuity design

Regression discontinuity designs can be used in cases where the assignment variable determines treatment either deterministically or probabilistically. ‘Sharp’ regression discontinuity refers to situations, in which the assignment variable determines treatment deterministically (Fig. 2a). When the continuity assumption is met and there is exchangeability between patients above and below the threshold, the difference in the means at the threshold can be calculated, which can be interpreted as the average causal effect (ACE) in the population near the threshold in sharp regression discontinuity designs. In this case, patients just below the threshold are unconditionally exchangeable with those just above the threshold. The ACE on the outcome, *Y*, in a sharp regression discontinuity design is thus identified at the threshold, *c*:

1 $$ \mathrm{ACE}={ \lim}_{z\uparrow c}E\left[{Y}_i\Big|{Z}_i=z\right]-{ \lim}_{z\downarrow c}E\left[{Y}_i\Big|{Z}_i=z\right] $$

Regression discontinuity designs can also be used in cases where treatment is probabilistically assigned by the threshold. This can occur in situations where treatment assignment is only partially determined by the assignment variable. This scenario is called ‘fuzzy’ regression discontinuity (Fig. 2b). In fuzzy regression discontinuity, the ACE can be conceptualized as similar to the ITT estimated in a randomized controlled trial (the effect of the randomized treatment). In the regression discontinuity case, the “ITT” (RD-ITT) is the effect of presenting just below the threshold compared to just above the threshold. The conceptual trial that the regression discontinuity design is emulating would be a trial in which treatment was randomly allocated for individuals in a narrow range around the threshold. In the absence of investigator-initiated randomization, the threshold itself serves as the randomization algorithm for the individuals around the threshold. In the fuzzy regression discontinuity design, the ITT can be interpreted as the effect of the threshold on outcomes for patients who present close to the threshold. Because this effect can concern the effect of a policy, such as the CD4 count at which a patient becomes eligible for immediate ART, the RD-ITT identified in fuzzy regression discontinuity is commonly viewed as the effect of interest from a policy perspective. In the CD4 count example, the regression discontinuity analysis would yield evidence of the effect of immediate eligibility for treatment compared to monitoring for eligibility and treatment initiation at a later time point, for individuals who are presenting to care with CD4 counts close to the threshold [12•].

### Regression Discontinuity with Non-Randomized Assignment Variables

Regression discontinuity designs can be viewed as analogous to randomized controlled trials in some circumstances when the assignment variable is non-randomized. When the assignment variable is measured with random error, the continuity assumption is expected to hold for patients near the threshold [12•]. Random measurement error is commonly found in applications in epidemiology. An example of an assignment variable measured with random error is CD4 count, which is used to determine ART eligibility among HIV-positive patients [15, 16]. In addition to random measurement error, CD4 counts are affected by exogenous factors, such as exercise [17], exposure to diesel exhaust [18], and smoking [19]. These factors result in substantial variability in CD4 count measurement. When the variability is exogenous to the causal structure under study, whether or not a patient presents immediately below (and thus is eligible for immediate treatment) or immediately above (and thus ineligible for immediate treatment) is random with respect to the causal structure, resulting in exchangeability in patients who are near the threshold. In this scenario, the assignment variable mimics a random variable for patients who are close to the threshold, allowing for estimation of causal effects. Importantly, unlike in the randomized controlled trial, where the causal effect is identified for the entire patient population, in the regression discontinuity design, the causal effect is only identified at the threshold (unless additional assumptions are evoked) [20].

**Fig. 3.**
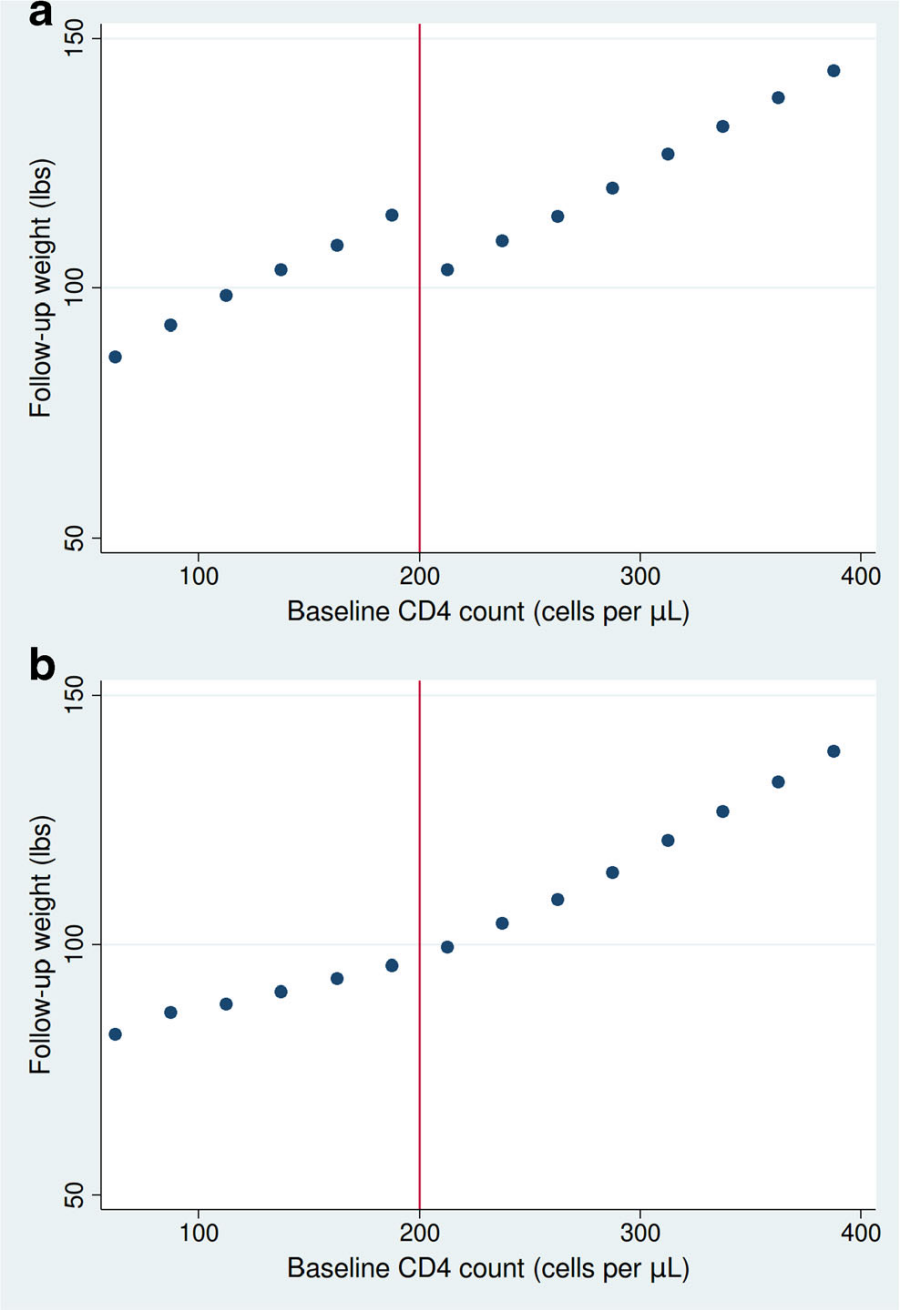
Visualizing treatment effects in regression discontinuity analyses. Illustration of the probability of outcome in the presence (**a**) and absence (**b**) of a treatment effect in a hypothetical scenario. In the case of a treatment effect, a discontinuity in probability of outcome can be visually seen at the threshold, whereas no discontinuity is seen, when there is no treatment effect

As part of a regression discontinuity analysis, evidence of a treatment effect can be easily visualized. Figure 3a demonstrates a scenario, in which there is a treatment effect. Evidence of the discontinuity in outcomes is evident at the threshold. Conversely, Fig. 3b demonstrates a scenario in which the effect is null. Notably, there is no discontinuity in outcomes at the threshold in this scenario.

In practice, identifying effects immediately at the threshold is infeasible in most scenarios, as few subjects will be immediately above or below the threshold. To estimate effects, information from patients on each side of the threshold must be used. This can be done by fitting a regression model with the form:

2 $$ E\left[{Y}_i\Big|{Z}_i\right]={\beta}_0+{\beta}_11\left[{Z}_i<c\right]+{\beta}_2\left({Z}_i-c\right)+{\beta}_3\left({Z}_i-c\right)*1\left[{Z}_i<c\right] $$

where *β*_1_ is the difference in the cutoff (equivalent to the effect of eligibility for treatment as defined by the threshold), *β*_2_ is the slope of the line below the threshold, and *β*_2_ + *β*_3_ is the slope of the line above the threshold. In order to validly estimate causal effects, it is thus necessary to correctly specify the functional form for the outcomes as a function of the assignment variable *Z*. Typically, models are presented with a range of bandwidths around the threshold [11•]. As the bandwidths get wider, more patients are included in the analysis, and the analysis will have greater statistical power. However, the importance of correctly specifying the functional form is greater at wider bandwidths compared to narrower bandwidths. Although these models can ‘borrow’ information from individuals further from the threshold and improve statistical power, it is important to correctly adjust for residual confounding by the assignment variable. Of note, even in models using wider bandwidths, the interpretation of the effect is still local to individuals who are close to the threshold. A bias-variance trade-off exists in selection of bandwidths, where wider bandwidths will be more powerful at the expense of increased risk of bias, and narrower bandwidths are less powerful but are likely to be less biased. Models using larger bandwidths often assess robustness through the inclusion of higher-order polynomials or splines, but effect size estimates are increasingly sensitive to model specification as bandwidth increases.

The assumptions for causal identification with regression discontinuity are summarized in Table 1. Whereas in the case where the assignment variable is randomly generated by the researcher the continuity (or exchangeability) assumption should hold if randomization were successful, in regression discontinuity designs with non-randomized assignment variables, evaluation of the plausibility of the continuity assumption is particularly important. In practice, this can be done by demonstrating that the assignment variable is continuous at the threshold (for example, by demonstrating in a histogram that there is no evidence of manipulation of the assignment variable at the threshold) and by demonstrating balance of covariates at baseline. Balance can be visually examined by presenting the distribution of baseline covariates by the continuous assignment variable. Such tests fulfill the same function as balance tests in randomized controlled trials.

**Table 1:** Assumptions of the regression discontinuity design

Assumption	Tests of assumptions
Assignment variable is measured continuously	•Verification that the assignment variable is measured and reported continuously •Knowledge that outcomes are measured for subjects regardless of whether or not they received treatment •Verification of how treatment is assigned to patients
Continuity of the assignment variable at the threshold	•Check for potential manipulation with a histogram of the assignment variable •Bunching at the threshold could indicate possible manipulation
Exchangeability (*the continuity assumption*)	•Covariate balance tests to demonstrate a balance of baseline covariates above and below the threshold •Plots of baseline covariates around the threshold to demonstrate that there is no discontinuity in factors that may be correlated with the assignment variable •Inclusion of baseline covariates as sensitivity analysis in the regression discontinuity model. These models should have point estimates that are similar to models without baseline covariates if the continuity of potential outcomes assumption holds
Consistency	•Assessment of how well defined the exposure of interest is
Positivity	•Ensure that there are individuals both above and below the threshold in the population
No misspecification of the functional form of the assignment variable	•Robustness checks, including flexible functional forms for the assignment variable, especially for models with wider bandwidths around the threshold

The two additional identifying assumptions required for causal inference include consistency and positivity. The consistency assumption is that an individual’s potential outcome had they received the treatment they actually received is equal to the individual’s observed outcome [21, 22]. To ensure consistency, interventions must be well defined [23]. In regression discontinuity, this assumption is generally expected to be met, as interventions are assigned by an assignment variable and thus are not biological variables, such as body weight, which could be “assigned” via a variety of mechanisms [23]. While biological variables may serve as assignment variables (e.g., blood pressure or blood sugar), they are used to assign a treatment that is, by definition of being determined by an assignment variable, well defined. The second assumption, positivity, implies that the probability of each treatment level is nonzero for each combination of treatment and confounder [24]. In the regression discontinuity design, the positivity assumption is met when there are individuals both above and below the threshold.

### Instrumental Variable Methods for Estimating Complier Average Causal Effects in Regression Discontinuity Designs

In the fuzzy regression discontinuity design, the effect of treatment  among those who take treatment because of the threshold rule can be identified using instrumental variable methods [25]. In this case, the threshold rule is an instrument for treatment. In the instrumental variable framework, the population of patients can be divided in to four latent subtypes: (1) the compliers, who are the subset of the population who follow the treatment regimen assigned to them by the threshold; (2) the defiers, who are the subset who do the opposite of what the threshold assigns them to; (3) the never takers, who do not take treatment regardless of which side of the threshold they are on; and (4) the always takers, who always take treatment regardless of what side of the threshold they are on. Instrumental variable methods can be used within the regression discontinuity framework to identify the causal effect among the compliers, known as the complier average causal effect (CACE), where the instrument is eligibility for treatment (i.e., the threshold). This effect is generalizable only to the compliers, although as a latent subtype, the subpopulation is not empirically identifiable [26]. Complier average causal effects can also be recovered in randomized controlled trials when there is noncompliance to the assigned treatment.

The CACE can be calculated in the regression discontinuity framework via the Wald instrumental variable estimator under identical identifying assumptions. The CACE can be estimated by scaling the effect of the threshold (RD-ITT) identified in Eq. 1 by the difference in the probability of treatment at the threshold (i.e., the probability of receiving treatment given presentation below vs. above the threshold). The instrumental variable estimator in the case of regression discontinuity can be defined as:

3 $$ \mathrm{CACE} = \frac{{ \lim}_{z\uparrow c}E\left[{Y}_i\Big|{Z}_i=z\right]-{ \lim}_{z\downarrow c}E\left[{Y}_i\Big|{Z}_i=z\right]}{{ \lim}_{z\uparrow c} Pr\left[{T}_i=1\Big|{Z}_i=z\right]-{ \lim}_{z\downarrow c} Pr\left[{T}_i=1\Big|{Z}_i=z\right]} $$

In the case of sharp regression discontinuity, the denominator of Eq. 3 is equal to 1, because the threshold assigns treatment perfectly. Thus, in sharp regression discontinuity, the RD-ITT is equivalent to the CACE and can be interpreted as the average causal effect. In fuzzy regression discontinuity, the CACE is generally expected to be further from the null than the RD-ITT, and the greater the difference between treatment assignment and actual treatment status, the greater the difference between the RD-ITT and the CACE. The significance of the effect of the threshold remains a valid test of the null hypothesis in CACE estimation using the instrumental variable estimator: if the RD-ITT is null, the CACE will be null as well.

The identification of the CACE requires several additional assumptions beyond those required for identification of the ITT in regression discontinuity designs. The assumptions are identical to those necessary in instrumental variable analyses [25]. First, the identified effect is the effect among the compliers (i.e., the effect among individuals who took treatment because of the threshold rule).

The identification of the CACE also requires the assumption of the exclusion restriction, i.e., the effect of the threshold (the instrument in the broader instrumental variable framework) only affects the outcome Y via actual treatment status T. The exclusion restriction is the fundamental assumption that underlies any instrumental variable analysis. In the randomized controlled trial case, randomization ensures that the exclusion restriction is met (if there is effective masking of treatment allocation). When the assignment variable is not randomized, the plausibility of the exclusion restriction can be evaluated via knowledge of the causal structure under study. In the regression discontinuity case, the exclusion restriction is expected to hold for individuals who are near the threshold. When wider bandwidths are used, the exclusion restriction will hold conditional on the assignment variable, when the assignment variable is included in the regression model. Of note, the exclusion restriction is not required for the calculation of the RD-ITT (the effect of the threshold on outcomes). The RD-ITT represents a population effect of the threshold itself. Although, in the fuzzy regression discontinuity case, some individuals may not have taken treatment as assigned by the threshold, the RD-ITT is simply the population effect of the threshold. It is only when calculating the CACE – i.e., when we are attributing the effect of the threshold to the effect of treatment itself – that the exclusion restriction must be met.

Finally, the monotonicity assumption must hold: No patients who would have taken up treatment if ineligible would not take up treatment if eligible (i.e., there are no defiers in the population). As a latent subtype, the population of patients who would be defiers is not identifiable. This assumption is therefore not empirically verifiable; however, knowledge of the causal structure may elucidate whether or not the monotonicity assumption is likely met.

### Tests for Determination of the Optimal Bandwidth

Recent work has described data-driven tests for selection of the optimal bandwidth for the regression discontinuity design [27, 28]. These methods are meant to assist the researcher with objective selection of the optimal bandwidth for their application, considering the bias-variance trade-off discussed above, rather than relying on arbitrary bandwidths. The objective of the optimal bandwidth estimators is to give researchers a reference point, from which they can assess the robustness of results to bandwidth variation in sensitivity analyses.

### Opportunities for Regression Discontinuity with Big Data

Regression discontinuity may offer opportunities for causal inference in the era of ‘big data’, i.e., data that is large in volume, arrives with high velocity, and is typically combined from a wide variety of sources. Data that are routinely collected, such as program monitoring data or electronic health data from national health systems, may provide opportunities for estimation of causal effects without randomized controlled trials, with the use of regression discontinuity. In clinical settings of routine data collection at large scale, measurement of potential assignment variables that determine treatment includes CD4 count for ART, intraocular pressure for glaucoma treatment, blood sugar for diabetes treatment, and blood pressure for antihypertensive treatment. Programmatically, routine monitoring data can sometimes be used to evaluate the causal impact of a novel intervention when intervention assignment is determined by thresholds, e.g., a date in calendar time or a minimum prevalence of a particular disease. Big datasets are particularly well suited to regression discontinuity analysis due to the large number of observations they are likely to contain. Because regression discontinuity must focus on the population near the relevant cutoff point, big data offers opportunities for sufficiently powered studies even with narrow bandwidth.

## Applications of the Regression Discontinuity Design in Epidemiology

Although application of the regression discontinuity design in epidemiology remains rare [11•], there have been several recent examples in the epidemiologic literature. Examples include the effect of relative younger age compared to their peers at entry to school on suicide [29], low-intensity telephone counseling on detection of metabolic syndrome [30], increased schooling on HIV status [31], human papillomavirus (HPV) vaccination on sexual behavior [32] and cervical dysplasia [33••], and the efficacy of prostate-specific antigen screening for detection of prostate cancer [10••]. These studies, reviewed below, report a range of practices in terms of discussion of the assumptions required for identification of causal effects and in their use of the CACE.

Matsubayashi and Ueda [29] report the effect of relative age in a grade (i.e., children who are on the younger end of the distribution of ages in a given grade) on suicide mortality. The authors utilize date of birth as the assignment variable and the school entry cutoff as the threshold rule. In expectation, individuals born just after the cutoff date (younger relative age) should have similar baseline characteristics to those just before the cutoff (older relative age). Although the authors report that the data generating process (exogenous variation in relative age due to the threshold) should result in a balance of baseline characteristics, a balance table is not presented. The authors report results for a range of bandwidths around the threshold (7 to 28 days). The authors discuss possible manipulation of the threshold by parents, getting their children into school earlier, but do not present a histogram of distribution of birth dates around the threshold. The CACE is not reported or discussed, and the results thus represent the effect of being born just before the cutoff date for starting school.

Yi et al. [30] present results for the effect of a telephone counseling intervention on untreated metabolic syndrome. Enrollees in the NHIS Metabolic Syndrome Management Programme were considered high risk if they had three or more metabolic syndrome criteria, whereas those with two or fewer were considered low risk. The authors divide the patient population into low- and high-risk groups and further subdivide the high-risk group into those who participated in the counseling intervention. Although this situation represents a fuzzy regression design, because not all enrollees in the high-risk group received the intervention, rather than calculating the CACE, an analysis comparing those who received the counseling session to the control was presented. This approach is identical to a per-protocol analysis in a randomized controlled trial, an analytic strategy that is known to suffer potential selection bias if there are factors associated with actual receipt of the intervention that are also associated with the outcome [34].

Behrman [31] reports the effect of primary schooling on adult HIV status among women in Malawi and Uganda. The author uses eligibility for the universal primary education (UPE) policy implemented in the nineties (age 13 or younger at policy implementation versus older) as the threshold variable and birth cohort as the assignment variable. The author discusses that this is a case of fuzzy regression discontinuity because there is grade repetition, meaning that some girls who are beyond the age of primary school will be exposed to UPE, as well as noncompliance, as some girls who are eligible for UPE will not attend school. The CACE, estimated using instrumental variable methods, is reported as the estimand of interest due to the fuzzy nature of the data, and the author discusses the exclusion restriction assumption. Sensitivity analyses are presented for a range of ages around the cutoff.

Smith et al. present the effect of HPV vaccination on cervical dysplasia and anogenital warts [33••] and sexual behaviors [32]. The assignment variable, birth date, was categorized into quarter years, and the threshold rule was birth on or after January 1, 1994 (eligible) versus earlier than January 1, 1994 (ineligible). The CACE was presented for each of the outcomes and calculated using instrumental variable methods in addition to the ITT. The authors discuss the differences in interpretation of the ITT and CACE but do not discuss the additional assumptions necessary for causal interpretation of the CACE.

Finally, Shoag et al. [10••] report a re-analysis of the Prostate Lung Colorectal and Ovarian (PLCO) trial focusing on the efficacy of prostate-specific antigen (PSA) screening for prostate cancer detection undertaken due to concerns about the high rate of PSA screening in the control arm. The authors used a regression discontinuity design in the screening arm of the trial to re-analyze the data, using a PSA of 4.0 ng/mL as the threshold for further prostate cancer workup (further screening) and PSA itself as the assignment variable. Using the regression discontinuity design in the screening arm of the trial, the authors were able to replicate the overall trial results that there was no decrease in prostate cancer-specific or overall mortality as a result of additional prostate cancer screening.

## Conclusions

Regression discontinuity designs offer the opportunity to identify causal effects in the absence of randomized controlled trials by taking advantage of an exogenous source of variation in treatment assignment induced by a threshold rule. This may offer advantages over traditional epidemiological methods, which adjust for observed covariates, in that the data generating process is expected to result in a balance of both observed and unobserved covariates at baseline. This data generating process can be conceptualized analogously to that of a trial, where the random variation in treatment assignment is investigator assigned, rather than due to another exogenous source. This framework facilitates interpretation of the effect of treatment eligibility and extensions to the CACE. As in a randomized controlled trial, the ITT may be interpreted as the effect of treatment assignment, and the CACE is the effect of treatment among the compliers.

Recent applications of regression discontinuity design demonstrate a variety of practices and room for improvement in analysis and reporting. Although several of the studies we reviewed included estimation of the CACE, thorough discussions of the additional assumptions required for this effect estimation were infrequently included. Furthermore, calculation of the per-protocol effect in the regression discontinuity framework is likely to suffer from selection bias, as in a randomized controlled trial. Restriction to the subgroup that adhered to treatment would lead to selection bias, if there were factors associated both with actual treatment status and the outcome in the causal structure. As a best practice, instrumental variable methods should be used for estimating the effect of treatment itself on outcomes. Researchers who include the CACE in their analyses should include a discussion of the exclusion restriction and its plausibility given the context and the particular treatment assignment rule under study, in addition to assessment of the other assumptions necessary for identification of the RD-ITT.

There are several important limitations to consider with any regression discontinuity design. Perhaps most importantly, effects from regression discontinuity designs can only be generalized to individuals with assignment variable values that are close to the threshold. When interpreting the results of regression discontinuity analyses, careful attention should be paid to the population to whom effects can be generalized. Regression discontinuity designs also require relatively large sample sizes to achieve adequate statistical power, due to the need to restrict the sample to individuals who are close to the threshold.

The studies described above demonstrate a variety of research questions that can be assessed using regression discontinuity designs, ranging from social to clinical exposures and outcomes. In randomized controlled trials, regression discontinuity designs can be used to complement results when there are issues with the internal validity of the primary analysis. In cohorts, regression discontinuity can generate strong causal evidence that may not be possible with other methodologies used for observational data. These analyses can yield causal evidence from real-world settings, without the artificiality often introduced through the processes in randomized controlled trials and thus with a high likelihood of external validity. Given the frequency with which threshold rules are used in clinical practice to determine treatment eligibility, regression discontinuity designs have the potential to greatly contribute to the generation of high-quality evidence in epidemiology.
